# TDOA-Based Target Tracking Filter While Reducing NLOS Errors in Cluttered Environments

**DOI:** 10.3390/s23094566

**Published:** 2023-05-08

**Authors:** Jonghoek Kim

**Affiliations:** System Engineering Department, Sejong University, Seoul 05006, Republic of Korea; jonghoek@gmail.com

**Keywords:** time difference of arrival, non-line-of-sight, NLOS error, NLOS error reduction, transmitter tracking, interacting multiple model kalman filters, time-efficient target tracking

## Abstract

We consider tracking a moving target in a wireless communication system that is based on the radio signal. Considering a bounded workspace with many unknown obstacles, we handle tracking a non-cooperative transmitter using multiple signal receivers. Here, a non-cooperative transmitter is a transmitter whose signal emission time is not known in advance. We consider a time difference of arrival (TDOA) location problem, which locates the transmitter by processing the signal measurement time at multiple receivers. In tracking a non-cooperative transmitter, non-line-of-sight (NLOS) errors occur if obstacles block the LOS line connecting the receiver and the moving transmitter. Our article addresses how to track a moving transmitter while decreasing the NLOS error in TDOA-only measurements. We propose an algorithm to localize a transmitter while decreasing the NLOS error in TDOA measurements. For tracking a moving transmitter in real time, we integrate the proposed localization algorithm and the interacting multiple model Kalman filter (IMM KF). As far as we know, our article is novel in tracking a moving transmitter based on TDOA-only measurements in an unknown mixed LOS/NLOS workspace. We show that the proposed filter considerably decreases the NLOS errors in TDOA-only measurements while running fast. Therefore, the proposed tracking scheme is suitable for tracking a moving transmitter in real time. Through MATLAB simulations, we show that the proposed filter outperforms other state-of-the-art TDOA filters, considering both time efficiency and tracking accuracy.

## 1. Introduction

Considering wireless communication systems, this article tackles the tracking of a non-cooperative transmitter moving in a bounded workspace with many obstacles that are not known in advance. Here, a non-cooperative transmitter indicates a transmitter whose signal emission time is not known in advance. This implies that the non-cooperative transmitter and the receiver are not synchronized with each other.

This paper considers a time difference of arrival (TDOA) problem, which localizes the transmitter by processing the signal measurement time at multiple signal receivers. TDOA has been widely applied for finding a non-cooperative transmitter location [1,2,3,4,5]. For instance, TDOA is applicable when multiple receivers are positioned to localize an RF emitter generating communication signals [6,7,8].

Using three or more receivers, TDOA algorithms locate a signal’s source from the different arrival times at the receivers [6,7]. TDOA requires accurate time synchronization among the receivers. A global positioning system (GPS) or intra-network can be used for accurate synchronization [6,9].

We tackle the tracking of a non-cooperative transmitter maneuvering in outdoor 2D environments with many unknown obstacles, such as mountains or buildings. As a transmitter moves, obstacles may block the line-of-sight (LOS) connecting the receiver and the transmitter. This blocking due to obstacles determines whether a receiver is in the LOS or not. The localization accuracy is severely affected by delayed signals propagated through non-line-of-sight (NLOS) paths. NLOS paths can be generated due to signals that are reflected on obstacles. In transmitter localization, harsh wireless propagation conditions due to obstacles, such as NLOS receivers, can result in highly biased estimates [10]. NLOS is the biggest error source in time-based location methods; thus, it is crucial to develop NLOS error mitigation algorithms in wireless communication systems to improve localization accuracy [11].

The following problem is tackled in our paper: *track a moving transmitter in unknown, cluttered environments while reducing NLOS errors in TDOA-only measurements*. To solve this problem, our article addresses the development of an algorithm to localize a transmitter while decreasing NLOS error in TDOA-only measurements. Then, we integrate the localization algorithm and Kalman filters [12] for tracking a moving transmitter in unknown, cluttered environments.

Our article considers a scenario where TDOA-only measurements may include NLOS errors. TDOA measurements without considering NLOS error cannot be applied directly as measurements in tracking filters (e.g., particle filters or Kalman filters [12]) since TDOA measurements may include NLOS errors. Furthermore, while the transmitter maneuvers in unknown, cluttered environments, LOS receivers can become NLOS receivers, and vice versa. Therefore, tracking a moving transmitter while decreasing NLOS error in TDOA-only measurements, is not a trivial task.

Several manuscripts [13,14,15,16] have tackled the tracking of a moving transmitter under TDOA-only measurements. The authors of [13] applied particle filters (PFs) for this task. However, PFs are not desirable since their computational load increases significantly as the number of particles increases. For tracking a moving transmitter, [14] developed a recursive least-squares (RLS) algorithm that smooths successive stationary transmitter estimations computed from the maximum likelihood. References [15,16] applied interacting multiple model Kalman filters (IMM KFs) for the tracking of a moving transmitter under TDOA measurements. However, the papers in this paragraph did not consider a scenario where TDOA measurements may include NLOS error. In practice, obstacles in the environment can generate NLOS receivers.

IMM KFs are suitable for tracking a moving transmitter in real time since IMM KFs are computationally efficient. IMM KFs can be used for tracking a moving target in environments where the global positioning system (GPS) signal is occasionally lost [17]. Hence, our article applies IMM KFs in [18,19,20] for tracking a moving transmitter while mitigating NLOS errors. MATLAB simulations showed that, as we applied the IMM KF, the RMSE decreased compared to the case where the IMM KF was not applied.

We propose an algorithm to localize a transmitter while decreasing the NLOS error in TDOA measurements. For tracking a moving transmitter, it is beneficial to consider the dynamics of the transmitter. Hence, we integrated the proposed localization algorithm and the IMM KF for tracking a moving transmitter in real time. In this way, we solved the problem of tracking a moving transmitter while decreasing the NLOS error in TDOA-only measurements. To the best of our knowledge, our paper is novel in tracking a moving transmitter based on TDOA-only measurements in an unknown mixed LOS/NLOS workspace.

The proposed tracking filter decreases NLOS errors considerably while running fast. Thus, the proposed filter can be applied to track a moving transmitter in real time. The effectiveness of the proposed tracker is verified by comparing it with state-of-the-art localization filters utilizing MATLAB simulations. Through MATLAB simulations, we show that the proposed filter outperforms other state-of-the-art TDOA filters, considering both time efficiency and localization accuracy.

The organization of our paper is as follows: Section 2 addresses the literature review of our article. Section 3 addresses preliminary information related to our study. Section 4 addresses the proposed tracking filter, which decreases NLOS errors in TDOA-only measurements. Section 5 addresses the MATLAB simulations used to demonstrate the proposed methods. Section 6 provides conclusions.

## 2. Literature Review

Many studies have handled how to decrease NLOS error in transmitter localization [21,22,23,24,25,26]. Many studies have discussed decreasing NLOS error in TDOA localization [7,27,28,29,30]. References [30,31,32,33] assumed that NLOS error models are available for localizing a transmitter. Reference [34] assumed that the NLOS error has bounded supports. However, modeling the NLOS error distribution or accessing the NLOS error bounds are not trivial since the NLOS error may change depending on obstacle materials or obstacle shapes.

In order to identify NLOS errors for a high-resolution wireless localization system, [35] addressed an NLOS classifier utilizing a machine learning algorithm called AdaBoost. The authors of [36] proposed an indoor TDOA-based 3D positioning approach with NLOS identification by machine learning. The authors of [36] discussed the correlations between measured distances based on TDOA, which have different performances in NLOS/LOS scenarios. However, machine learning approaches may not work well in cases where we do not have sufficient training samples or biased training data are used. Furthermore, training a large number of samples may be computationally heavy. Machine learning approaches assume that obstacles in the environment are known in advance. However, this assumption does not hold in our paper. Note that our article does not depend on machine learning techniques, and our manuscript handles tracking a transmitter in outdoor 2D environments with many unknown obstacles, which are distinct from known indoor environments.

We considered the general case where modeling the NLOS error distribution is not feasible. Various papers have addressed NLOS error reduction methods without utilizing NLOS error models [3,7,27,33,37,38,39,40]. Under the assumption that obstacle information is known a priori, [40] addressed NLOS error reduction methods for TDOA measurements without utilizing NLOS error models. However, obstacle information may not be available in practice. Therefore, our article handles the general case where obstacle information is not available.

Reference [27] addressed how to detect NLOS measurements by utilizing the time history of its range measurements. The authors of [11] addressed how to mitigate NLOS errors in TDOA measurements under the assumption that a single LOS receiver is known in advance. Assuming that a single LOS receiver is known in advance, [29] addressed how to identify NLOS receivers utilizing the defined TDOA residual. However, the assumption of a known LOS receiver is too strong in practice. A receiver selection scheme (RSS) has been developed for finding LOS receivers among all receivers [7]. This scheme is to iteratively choose three receivers among all receivers, then calculate the transmitter estimate utilizing the three receivers. However, this RSS is not effective in cases where the number of LOS receivers is much larger than three.

Time of arrival (TOA) measurements have been widely applied for localizing a cooperative transmitter. Considering TOA measurements, references [3,33,37,38,39] introduced NLOS error reduction strategies that do not rely on NLOS error models. In our paper, TDOA measurements are different from TOA measurements since TDOA measurements consider cases where the signal emission time is not known to any receiver [38,39,41].

Considering the TDOA problem where NLOS error distribution is not available, the authors of [42] addressed a transmitter estimate approach that transforms a TDOA architecture into a TOA architecture, together with a semidefinite programming (SDP) approach with new constraints. Considering the case where the node location observation error is large, [43] developed the maximum likelihood formulation of the TDOA localization problem and provided SDP relaxations for this problem. The authors of [44] applied a data-selective approach and proposed a closed-form least-squares solution disregarding poor measurements. Reference [44] utilized two objective functions, one to calculate an estimated solution and the other one to test that particular solution.

An SDP approach in [42,43] utilized the optimization tool, and its computational load was much higher than non-optimization methods. Through MATLAB simulations, we show that the computational load of [42,43] is too heavy and thus not suitable for real-time target tracking.

We propose a fast algorithm to localize a transmitter while decreasing NLOS error in TDOA-only measurements. We show the superiority of the proposed tracking method by comparing it with [42,43,44] through MATLAB simulations. Through MATLAB simulations, we show that the proposed filter outperforms [42,43,44] considering both time efficiency and localization accuracy.

As far as we know, our paper is novel in tracking a moving transmitter based on TDOA-only measurements in an unknown mixed LOS/NLOS workspace. For tracking a moving transmitter, it is beneficial to consider the transmitter’s dynamics as we localize the transmitter. Hence, we integrated the proposed localization algorithm and the IMM KF for tracking a moving transmitter in real time. In this way, we solved the problem of tracking a moving transmitter while decreasing the NLOS error in TDOA-only measurements. MATLAB simulations show that as we applied the IMM KF, the RMSE decreased compared to the case where the IMM KF was not applied.

## 3. Preliminary Information

This article tackles tracking a moving transmitter in outdoor 2D environments with many unknown obstacles, such as mountains or buildings. Suppose *N* receivers are deployed to measure the arrival time of a signal emitted from an unknown transmitter. We assume that we can localize the position of every receiver in global coordinate systems and that communication links among all receivers are established. A GPS or an intra-network can be used for accurate synchronization [6,9]. Considering cluttered environments with NLOS errors, this article tackles the problem of tracking a non-cooperative transmitter utilizing the TDOA information of each receiver whose global position is known in advance.

### 3.1. Time Difference of Arrival (TDOA)

We address the TDOA problem considered in our article. Let $r_{i}$(i∈{1,2,…,N}) denote the *i*-th receiver. Let $r_{i}$ define the 2D location of $r_{i}$. Let $\left(x_{i},y_{i}\right)$ define the 2D location of $r_{i}$. We assume that $\left(x_{i},y_{i}\right)$ is known in advance. The unknown transmitter location is (x,y).

Let *C* define the speed of the signal generated from the transmitter. Let $t_{i}$ define the signal reception time at the receiver $r_{i}$. Since the non-cooperative transmitter and the receiver are not synchronized, $t_{i}$ is not known a priori. We define $R_{r_{i}}$ as

(1) $$\begin{array}{c} R_{r_{i}}=C\times t_{i}. \end{array}$$

Considering the noise in the signal reception time $t_{i}$, the equation for $R_{r_{i}}$ is rewritten as

(2) $$\begin{array}{c} R_{r_{i}}=\left(\sqrt{{\left(r_{i}\left[1\right]-x\right)}^{2}+{\left(r_{i}\left[2\right]-y\right)}^{2}}\right)+n_{i}, \end{array}$$

where $n_{i}$ defines the distance error caused by the noise in the signal reception time $t_{i}$. In Equation (2), $r_{i}\left[l\right]$ denotes the *l*-th element in $r_{i}$.

Consider the LOS scenario, where the LOS line connecting the transmitter and $r_{i}$ is not blocked by obstacles. We consider the case where each receiver is identical and unbiased. In LOS environments, the measurement noise $n_{i}$ in Equation (2) has a Gaussian distribution with zero mean and variance $\sigma^{2}$ that is not known in advance. In LOS environments, $n_{i}$ can be generated due to thermal noise [11]. Increasing the signal-to-noise ratio (SNR) can reduce σ [11].

Consider the NLOS scenario, where the LOS line connecting the transmitter and $r_{i}$ is blocked by obstacles. In this NLOS scenario, $n_{i}$ in Equation (2) indicates the NLOS error due to the signal blocking event. The modeling of the NLOS error distribution or accessing the NLOS error bound are not trivial tasks, since the NLOS error may change depending on obstacle materials or obstacle shapes. We thus assume that the NLOS error model is not known in advance. The NLOS is the biggest error source in time-based location methods; thus, it is crucial to develop NLOS error mitigation algorithms in wireless communication systems to improve localization accuracy [11].

Since the signal emission time is not accessible, a receiver $r_{i}$ cannot directly measure $t_{i}$ in Equation (1). Since all receivers are synchronized, the cross-correlation between two receivers, $r_{i}$ and $r_{j}$, can be applied to determine the TDOA $t_{i}-t_{j}$. In practice, identical signals reach the receiver at different times, due to multipath effects. In this case, we use the “signal of first arrival” for deriving the TDOA $t_{i}-t_{j}$.

Once the TDOA $t_{i}-t_{j}$ is measured, $C\times (t_{i}-t_{j})$ builds a hyperbolic curve indicating feasible transmitter locations. The TDOA range equation related to (x,y) is

(3) $$\begin{array}{ccc} R_{r_{i},r_{j}} & = & R_{r_{i}}-R_{r_{j}}. \end{array}$$

Here, recall that $R_{r_{i}}$ is defined in (1).

Under Equation (2), Equation (3) leads to

(4) $$\begin{array}{c} R_{r_{i},r_{j}}=∥\left(r_{i}\left[1\right]-x,r_{i}\left[2\right]-y\right)∥-∥\left(r_{j}\left[1\right]-x,r_{j}\left[2\right]-y\right)∥+n_{i}-n_{j}. \end{array}$$

A 2D transmitter location can be computed from the intersection of two or more hyperbolas generated from three or more TDOA measurements [7]. In the case where we only have two LOS receivers, TDOA generates a single hyperbola, and any point on the hyperbola can be a transmitter solution. References [6,7] thus stated that one needs at least three LOS receivers to locate a transmitter utilizing any TDOA algorithm.

### 3.2. Kalman Filter (KF)

This subsection briefly discusses the KF, which is applied for tracking a transmitter in our article. The references [12,45] address detailed explanations of KF. We omit the detailed explanation of the KF. Considering linear systems, the state-space models for the KF are as follows:

Linear process model:

(5) $$\begin{array}{c} X_{k+1}=FX_{k}+m_{k}. \end{array}$$

Linear measurement model:

(6) $$\begin{array}{c} z_{k}=HX_{k}+n_{k}. \end{array}$$

Here, $X_{k}$ defines the state vector at sample-stamp *k*. In (5) and (6), F and H are constant matrices since we consider a linear process model and a linear measurement model. In addition, $m_{k}$ and $n_{k}$ are process noise and measurement noise, respectively.

In (5), we assume that the process noise $m_{k}$ has a normal distribution with mean 0 and the error covariance matrix $Q_{k}$. In (6), we assume that the measurement noise $n_{k}$ has a normal distribution with mean 0 and the error covariance matrix $R_{k}$. We further assume that the process noise $m_{k}$ and measurement noise $n_{k}$ are not correlated to each other.

The KF calculates the estimate of $X_{k}$ and its covariance matrix at each sample-stamp *k*. In the KF, ${\hat{X}}_{k|k}$ is the estimate at sample-stamp *k*, and $P_{k|k}$ is the covariance matrix of ${\hat{X}}_{k|k}$. The procedure has the following two updates: the prediction and the measurement update.

#### 3.2.1. Prediction

Suppose that the current sample-stamp is k−1. We predict the state vector at sample-stamp *k* under

(7) $$\begin{array}{c} {\hat{X}}_{k|k-1}=F{\hat{X}}_{k-1|k-1}. \end{array}$$

In addition, we predict the error covariance matrix under

(8) $$\begin{array}{c} P_{k|k-1}=FP_{k-1|k-1}F^{T}+Q_{k-1}. \end{array}$$

#### 3.2.2. Measurement Update

The Kalman gain is calculated as

(9) $$\begin{array}{c} K_{k}=P_{k|k-1}H^{T}{\left(HP_{k|k-1}H^{T}+R_{k}\right)}^{-1}. \end{array}$$

Both the state vector and its error covariance matrix are updated under

(10) $$\begin{array}{c} {\hat{X}}_{k|k}={\hat{X}}_{k|k-1}+K_{k}\left(z_{k}-H{\hat{X}}_{k|k-1}\right). \end{array}$$

(11) $$\begin{array}{c} P_{k|k}=\left(I-K_{k}H\right)P_{k|k-1}{\left(I-K_{k}H\right)}^{T}+K_{k}R_{k}K_{k}^{T}. \end{array}$$

## 4. Transmitter Tracking While Decreasing NLOS Error in TDOA Measurements

In locating a 2D transmitter, the location error increases as obstacles block the LOS line connecting the receiver and the transmitter. The error due to NLOS environments is called *NLOS error*. This section addresses how to track a moving transmitter while decreasing the NLOS error in TDOA-only measurements.

### 4.1. System Models

This subsection introduces the process model and the measurement model in our transmitter tracking approach. In our problem, the process model is Equation (5) with $X_{k}={\left[x_{k},y_{k},v_{x,k},v_{y,k}\right]}^{T}$. Here, $\left[x_{k},y_{k},v_{x,k},v_{y,k}\right]$ indicates the location and the velocity of the transmitter at each sample-stamp *k*.

The measurement model is Equation (6) with $H=\left(\begin{array}{cccc} 1 & 0 & 0 & 0\\ 0 & 1 & 0 & 0 \end{array}\right)$. In Equation (6), $z_{k}$ is the transmitter’s location measurement at sample-stamp *k*. $z_{k}$ is called the *TDOA transmitter estimate*, since $z_{k}$ is computed utilizing TDOA measurements at sample-stamp *k*.

Section 4.4 shows how to compute $z_{k}$ while decreasing NLOS error. In Section 4.4, $\hat{E}$ in Equation (23) is set as the location measurement $z_{k}$ in Equation (6). The IMM KF in Section 4.5 utilizes Equation (6) as its measurement equation in order to compute the transmitter estimate at each sample-stamp *k*.

### 4.2. Definitions and Assumptions

This subsection addresses several definitions and assumptions. Among *N* receivers, the *LOS receiver* is a receiver such that the LOS line connecting the receiver and the transmitter is not blocked by obstacles.

We consider a general case where reliable modeling of NLOS error is not feasible. We have the following assumptions.

- (A1) Considering unknown, cluttered environments, at least three LOS receivers exist among all receivers. However, these LOS receivers are not known in advance.
- (A2) Considering LOS receivers, the measurement noise $n_{i}$ in Equation (2) has a Gaussian distribution with zero mean and variance $\sigma^{2}$ that is not known in advance.
- (A3) The transmitter exists inside a bounded workspace, whose boundary is known in advance.

References [6,7] mention that at least three LOS receivers are necessary to localize a transmitter utilizing any TDOA algorithm. Therefore, any TDOA algorithm requires Assumption (A1).

We consider the case where each receiver is identical and unbiased. The references [30,31,46] applied Assumption (A2).

### 4.3. Least-Squares Estimation (LSE) for Solving the TDOA Localization

Let *I* define a receiver set with ∥I∥ receivers, say $r_{1}^{I}$,$r_{2}^{I}$,…,$r_{∥I∥}^{I}$. We introduce how to compute the transmitter estimate utilizing TDOA measurements of these receivers.

Recall that Equation (3) addressed the equation for the range measurements in TDOA problems. Assigning $r_{1}^{I}\in I$ as the reference, the range measurement equation in Equation (3) without measurement noise becomes

(12) $$\begin{array}{c} R_{r_{i}^{I},r_{1}^{I}}=R_{r_{i}^{I}}-R_{r_{1}^{I}}. \end{array}$$

Here, i∈{2,3,…,∥I∥}, and we use

(13) $$\begin{array}{c} R_{r_{i}^{I}}=∥\left(r_{i}^{I}\left[1\right]-x,r_{i}^{I}\left[2\right]-y\right)∥. \end{array}$$

We define $z={\left(x-r_{1}^{I}\left[1\right],y-r_{1}^{I}\left[2\right],R_{r_{1}^{I}}\right)}^{T}$. Let $\hat{z}$ define the estimate of z. In addition, let

(14) $$\begin{array}{c} \hat{Z}={\left(\hat{z}\left[1\right]+r_{1}^{I}\left[1\right],\hat{z}\left[2\right]+r_{1}^{I}\left[2\right]\right)}^{T} \end{array}$$

define the 2D *transmitter estimate*.

Least squares estimation (LSE) in [47] is applied to compute $\hat{z}$ in TDOA problems. Equation (12) leads to

(15) $$\begin{array}{c} G\hat{z}=h, \end{array}$$

where

(16) $$\begin{array}{c} G=\left(\begin{array}{ccc} r_{2}^{I}\left[1\right]-r_{1}^{I}\left[1\right], & r_{2}^{I}\left[2\right]-r_{1}^{I}\left[2\right], & R_{r_{2}^{I},r_{1}^{I}}\\ \vdots & \vdots & \vdots \\ r_{∥I∥}^{I}\left[1\right]-r_{1}^{I}\left[1\right], & r_{∥I∥}^{I}\left[2\right]-r_{1}^{I}\left[2\right], & R_{r_{∥I∥}^{I},r_{1}^{I}} \end{array}\right). \end{array}$$

Let G[i,j] define the element of G at the *i*-th row and *j*-th column. In Equation (15), h is

(17) $$\begin{array}{c} h=0.5\left(\begin{array}{c} {\left(G\left[1,1\right]\right)}^{2}+{\left(G\left[1,2\right]\right)}^{2}-{\left(G\left[1,3\right]\right)}^{2}\\ \vdots \\ {\left(G\left[∥I∥,1\right]\right)}^{2}+{\left(G\left[∥I∥,2\right]\right)}^{2}-{\left(G\left[∥I∥,3\right]\right)}^{2} \end{array}\right). \end{array}$$

See [47] for detailed derivations of Equation (15). The LSE solution in Equation (15) is computed utilizing

(18) $$\begin{array}{c} \hat{z}={\left(G^{T}G\right)}^{-1}G^{T}h. \end{array}$$

Then, by applying Equation (18) into Equation (14), we compute the transmitter estimate $\hat{Z}$. Acknowledge that the LSE solution in Equation (18) ignores the measurement noise. However, considering the computational load, the simple LSE solution in Equation (18) is desirable.

### 4.4. NLOS Error Reduction Algorithm

Since the NLOS is the biggest error source in time-based location methods, we must develop NLOS error mitigation algorithms to improve localization accuracy [11]. This subsection addresses how to decrease the NLOS error in the TDOA problem. Our NLOS error reduction approach is to find a feasible LOS receiver set (each set has at least three receivers) that is most probable to consist of LOS receivers.

Note that any TDOA algorithm requires Assumption (A1). Under Assumption (A1), we iteratively increase the number of feasible LOS receivers, starting from three, and calculate the transmitter estimate utilizing each feasible LOS receiver set.

Let *K* define the number of feasible LOS receivers in each receiver set. This implies that each set consists of *K* receivers. Since one has *N* receivers in total, the number of total receiver sets is $C_{N}^{K}$. Let $I_{c}$, where $c\in \{1,2,\ldots ,C_{N}^{K}\}$, define a receiver set, such that each set has *K* receivers.

For notation convenience, let $I_{c}^{i}$ define the *i*-th receiver in $I_{c}$. Here, i∈{1,2,…,K} in $I_{c}^{i}$, since each receiver set has *K* receivers. In addition, let $I_{c}^{i}$ define the 2D location of $I_{c}^{i}$ (i∈{1,2,…,K}).

Since the number of true LOS receivers may be bigger than three, we keep updating *K* and iterate the algorithm utilizing the updated *K*. *K* is updated in the following order: (N−3)→3→(N−4)→4→(N−5)→…. This order is defined as the *ReceiverSelectOrder*.

Considering the computational burden, we set the limit for the update in *K*. If *K* becomes a limit, say $K_{limit}$, then *K* is not updated any more. In MATLAB simulations (Section 5), *K* is updated in the following order: $\left(N-3\right)\to 3\to \left(N-4\right)\to K_{limit}=4$.

Consider a receiver set $I_{c}$, such that each set has *K* receivers. We compute ${\hat{Z}}_{c}$ by applying the LSE solution (Equation (18)) to all receivers in $I_{c}$. We calculate the associated $\bar{Res}$, which is defined as follows.

(19) $$\begin{array}{c} \bar{Res}\left(I_{c}\right)=\frac{Res(I_{c})}{K}, \end{array}$$

Here, $Res(I_{c})$ is

(20) $$\begin{array}{c} Res\left(I_{c}\right)=\sum_{I_{c}^{j}\in I_{c}}\frac{{\left(R_{I_{c}^{j},I_{c}^{1}}-{\hat{I}}_{c}^{j}+{\hat{I}}_{c}^{1}\right)}^{2}}{{\left(C\sigma \right)}^{2}}, \end{array}$$

where

(21) $$\begin{array}{c} {\hat{I}}_{c}^{j}=∥(I_{c}^{j}\left[1\right]-{\hat{Z}}_{c}\left[1\right],I_{c}^{j}\left[2\right]-{\hat{Z}}_{c}\left[2\right])∥, \end{array}$$

and

(22) $$\begin{array}{c} {\hat{I}}_{c}^{1}=∥\left(I_{c}^{1}\left[1\right]-{\hat{Z}}_{c}\left[1\right],I_{c}^{1}\left[2\right]-{\hat{Z}}_{c}\left[2\right]\right)∥. \end{array}$$

In Equation (20), $R_{I_{c}^{j},I_{c}^{1}}$ defines the TDOA range measurement related to two receivers $I_{c}^{j}\in I_{c}$ and $I_{c}^{1}\in I_{c}$. See Equation (3) for the TDOA range equation. In addition, ${\hat{I}}_{c}^{j}-{\hat{I}}_{c}^{1}$ defines the estimated TDOA range as the transmitter is at ${\hat{Z}}_{c}$ and two receivers are at $I_{c}^{j}\in I_{c}$ and $I_{c}^{1}\in I_{c}$ respectively.

The proposed algorithm has the following steps.

1 Let $I_{0}=\left\{r_{1},r_{2},\ldots ,r_{N}\right\}$ define the set of all receivers. Initially, we set K=N−3.
2 From $I_{0}$, we compute all receiver sets, such that each receiver set has *K* receivers. The number of total receiver sets is $C_{N}^{K}$. Let $I_{c}$ define each receiver set. Using all receivers in each receiver set $I_{c}$, one calculates the transmitter estimate ${\hat{Z}}_{c}$ utilizing the LSE solution (Equation (18)) in Section 4.3. In addition, we calculate the associated $\bar{Res}$, as defined in Equation (19).
3 Under Equation (20), a reliable estimate has a smaller $\bar{Res}\left(I_{c}\right)$. Therefore, we find a receiver set with the minimum $\bar{Res}$. Let $I_{min}\in \left\{I_{1},I_{2},\ldots ,I_{C_{N}^{K}}\right\}$ define the found receiver set.
4 Let $∥I_{min}∥$ define the number of elements in $I_{min}$. From $I_{min}$, we compute all receiver sets, such that each receiver set has $∥I_{min}∥-1$ receivers. In this way, we build $∥I_{min}∥$ new receiver sets, $\left\{I_{c},C=1,2,\ldots ,∥I_{min}∥\right\}$. For the $∥I_{min}∥$ new receiver sets, one computes the transmitter estimate utilizing the LSE solution (Equation (18)) in Section 4.3. In addition, one utilizes Equation (19) to derive the associated $\bar{Res}$. Among the $∥I_{min}∥$ new receiver sets, one searches for the set with the minimum $\bar{Res}$. Let $I_{min}$ define the found receiver set.
5 If $∥I_{min}∥$ becomes 3, then jump to the next step. Else, jump to step [4].
6 Derive the fused estimate $\hat{E}$ utilizing Equation (23). If $K\neq K_{limit}$ and $\hat{E}$ is outside the bounded workspace, then update *K* under the ReceiverSelectOrder. Then, jump to Step [2]; else, this algorithm is finished, and we select $\hat{E}$ in Equation (23) as the algorithm output.

We explain the step [4] of the above algorithm. From $I_{min}$, we compute all receiver sets, such that each receiver set has $∥I_{min}∥-1$ receivers. In this way, we build $∥I_{min}∥$ new receiver sets, $\left\{I_{c},C=1,2,\ldots ,∥I_{min}∥\right\}$. For instance, suppose that $I_{min}=\left\{1,2,3,4\right\}$. Since $∥I_{min}∥=4$, new receiver sets are generated as $I_{1}=\left\{2,3,4\right\}$, $I_{2}=\left\{1,3,4\right\}$, $I_{3}=\left\{1,2,4\right\}$, and $I_{4}=\left\{1,2,3\right\}$.

In the last step of the above algorithm, we calculate the *TDOA estimate output* under the following equation.

(23) $$\begin{array}{c} \hat{E}=\frac{\sum_{k=1}^{V}{\hat{Z}}_{k}{\left(\bar{Res}\left(I_{k}\right)\right)}^{-1}}{\sum_{k=1}^{V}{\left(\bar{Res}\left(I_{k}\right)\right)}^{-1}}. \end{array}$$

Here, *V* defines the total number of transmitter estimates, while $∥I_{min}∥$ is updated from *K* to 3.

In Equation (23), the weight of an estimate ${\hat{Z}}_{k}$ is $\frac{1}{\bar{Res}\left(I_{k}\right)}$. Under Equation (20), a reliable estimate ${\hat{Z}}_{k}$ has a smaller $\bar{Res}\left(I_{k}\right)$. Therefore, in $\hat{E}$, a reliable estimate ${\hat{Z}}_{k}$ has a larger weight $\frac{1}{\bar{Res}\left(I_{k}\right)}$.

#### Exception Handling

In the MATLAB simulations (Section 5), we set $K_{limit}=4$. Suppose that *K* reached $K_{limit}$ and $\hat{E}$ derived under (23) is outside the bounded workspace. In this case, we need to find a solution that is inside the bounded workspace.

We randomly select several receivers and derive a feasible solution using the selected receivers. Under Assumption (A1), we update *K* in the following order: 3→4→⋯→N. This order is defined as the *SelectOrder*.

Associated with each *K* in the SelectOrder, we randomly select *K* receivers among all receivers. Then, the selected receivers are set as the elements in the receiver set associated to *K*. The SelectOrder has N−2 elements in total. Hence, while *K* is updated under the SelectOrder, we generate N−2 receiver sets in total.

Let $Q_{K}$, K∈{3,4,⋯,N}, define each receiver set derived utilizing the SelectOrder. Note that $∥Q_{K}∥$ is equal to *K*. For instance, suppose that N=6. We may have $Q_{3}=\left\{1,4,5\right\}$ and $Q_{4}=\left\{2,4,5,6\right\}$.

Using all receivers in $Q_{K}$, we calculate the transmitter estimate ${\hat{Z}}_{K}$ utilizing the LSE solution (Equation (18)) in Section 4.3. In addition, we calculate the associated $\bar{Res}$, as defined in Equation (19).

Among all $Q_{K}$, K∈{3,4,⋯,N}, we find a receiver set with the minimum $\bar{Res}$, such that its associated LSE solution ${\hat{Z}}_{K}$ is inside the bounded workspace. The found LSE solution ${\hat{Z}}_{K}$ is set as the TDOA estimate output, i.e., $\hat{E}$ in (23).

### 4.5. IMM KF

This subsection addresses the IMM KF used in our manuscript. The IMM KF applies multiple motions, such as coordinated turn (CT) motion and constant velocity (CV) motion, followed by merging the motions to estimate the transmitter’s state vector. In the IMM KF, three motions are applied: the CV motion, the CT motion (left turn), and the CT motion (right turn).

In the CV motion, Equation (5) utilizes F as

(24) $$\begin{array}{c} F=\left(\begin{array}{cccc} 1 & 0 & T & 0\\ 0 & 1 & 0 & T\\ 0 & 0 & 1 & 0\\ 0 & 0 & 0 & 1 \end{array}\right). \end{array}$$

Here, *T* is the sampling interval in a discrete-time system.

In the CT motion (right turn), F in Equation (5) is selected as

(25) $$\begin{array}{c} F=\left(\begin{array}{cccc} 1 & 0 & \frac{\sin(wT)}{w} & \frac{\left(\cos(wT)-1\right)}{w}\\ 0 & 1 & \frac{\left(1-\cos(wT)\right)}{w} & \frac{\sin(wT)}{w}\\ 0 & 0 & \cos(wT) & -\sin(wT)\\ 0 & 0 & \sin(wT) & \cos(wT) \end{array}\right). \end{array}$$

In Equation (25), *w* is the tuning parameter indicating the turn rate of the transmitter during the CT motion.

The IMM KF is applied for computing the estimate and its covariance at each sample-stamp *k*. The *model transition probability* is set as follows. The probability of the CV motion is $CV_{p}=0.8$, and that of any other motion is $\frac{1-CV_{p}}{2}$. In the IMM KF, ${\hat{X}}_{k|k}$ is the state estimate at sample-stamp *k*, and $P_{k|k}$ is the error covariance matrix of ${\hat{X}}_{k|k}$.

The IMM KF is composed of four steps: the interaction step, the filtering step, the model probability update, and combination. In the interaction step, the model state estimates and covariances are mixed utilizing conditional model probabilities. In the filtering step, the likelihood of each model is calculated utilizing the innovations derived during the state update. This step is finished after the state prediction of each mixed state estimate. After all filter models have been updated, the model probabilities are updated. The combined state estimate and covariance are calculated from the updated filtered states from each model, weighted by the updated model probabilities. Details of the IMM KF are discussed in [18,19,20].

Since each motion (CV or CT) is linear, each motion utilizes the prediction step of the KF [12]. The measurement equation used in the IMM KF is Equation (6) with $H=\left(\begin{array}{cccc} 1 & 0 & 0 & 0\\ 0 & 1 & 0 & 0 \end{array}\right)$. In Equation (6), $z_{k}$ is computed utilizing TDOA measurements at sample-stamp *k*. In other words, the TDOA estimate output $\hat{E}$ in Equation (23) is set as the location measurement $z_{k}$ in Equation (6).

Suppose that the IMM KF track is initiated at sample-stamp *k*. ${\hat{X}}_{k|k}$ is initiated under the rule-based track initiation technique in [48]. Recent $N_{t}$ TDOA estimate outputs are utilized to initiate the IMM KF. In addition, $P_{k|k}$ is initiated as

(26) $$\begin{array}{c} P_{k|k}=\mathrm{diag}\left({\left(t_{p}\right)}^{2},{\left(t_{p}\right)}^{2},\frac{V_{max}^{2}}{3},\frac{V_{max}^{2}}{3}\right), \end{array}$$

where $t_{p}$ is a tuning parameter indicating the uncertainty in TDOA measurements. Furthermore, $V_{max}$ is the transmitter’s maximum speed, which is assumed to be known a priori.

In some cases, $z_{k}$ may be far from the true transmitter position. Therefore, a TDOA measurement $z_{k}$ is discarded if the $z_{k}$ is NULL or if $z_{k}$ satisfies the following condition.

(27) $$\begin{array}{c} \mu_{k}^{T}{\left(HP_{k|k-1}H^{T}+R_{k}\right)}^{-1}\mu_{k}>\sum^{2}, \end{array}$$

where $\mu_{k}=z_{k}-H{\hat{X}}_{k|k-1}$ and ∑>0. Equation (27) implies that we discard a TDOA measurement whose location is too far from the current IMM KF estimate output. If $z_{k}$ is NULL or Equation (27) is satisfied, then we do not perform the measurement update and only perform the prediction step in the KF process.

In (27), $R_{k}$ is the covariance of $z_{k}$, indicating the variance of $z_{k}$. Curve fitting is applied to recent measurements in order to calculate $R_{k}$ adaptively. The transmitter’s true trajectory is conjectured utilizing the second-order polynomial curve. Then, the measurement error is conjectured utilizing the error between the curve and the measurements. For the adaptive adjustment of $R_{k}$, we use the adaptive adjustment method in [40].

## 5. MATLAB Simulations

This section demonstrates the performance of our transmitter tracking approach under MATLAB simulations. We consider locating a transmitter in unknown, 2D, cluttered environments with rectangular obstacles.

The simulation settings are as follows. The signal speed is $C=3\times 10^{8}$ m/s. For filter initialization, we apply the rule-based track initiation approach in [48]. Recent $N_{t}=4$ TDOA estimate outputs are applied for initiating the IMM KF. In Equation (26), we apply $t_{p}=300$ and $V_{max}=30$ m/s. The sampling interval is T=10 s.

**Figure 1:**
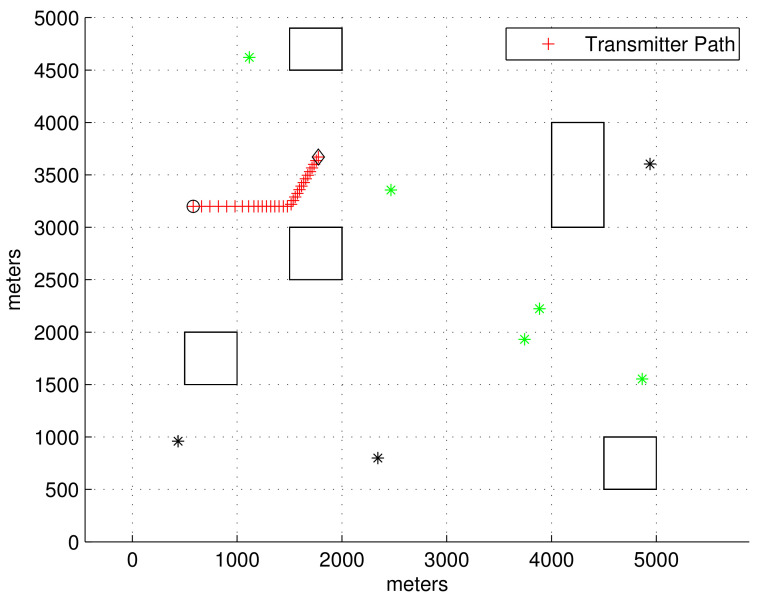
Scenario 1. The transmitter’s location at every sample-stamp is plotted with a red cross. The start point of the transmitter is marked with a black circle, and the end point of the transmitter is marked with a black diamond. Reflected signals can be generated due to obstacles, which are plotted with rectangles in the workspace. As the transmitter moves, an LOS receiver may become an NLOS receiver, and vice versa. At the moment when the simulation ends, LOS receivers are plotted with green asterisks, and NLOS receivers are plotted with black asterisks.

We set up the obstacle environment as plotted in Figure 1. Note that obstacles are plotted with rectangles in the workspace. Recall that an LOS receiver is a receiver such that the LOS line connecting the receiver and the transmitter is not blocked by obstacles. The signal arrival time for each LOS receiver is d/C+n, where *d* is the relative distance from the LOS receiver to the transmitter. In addition, *n* is a zero-mean Gaussian noise with a standard deviation *σ*.

The proposed localization approach does not require any prior information on NLOS error models. Hence, one can apply any distribution for NLOS error models. In numerical simulations, the signal arrival time for each NLOS receiver is randomly distributed in the interval [d/C+n+500/C, d/C+n+1000/C]. This implies that the distance of NLOS noise is in the interval [500, 1000] in meters. Note that while the transmitter moves, an LOS receiver may become an NLOS receiver, and vice versa.

In the IMM KF, the transmitter’s turn rate is selected as w=5 degrees per second in Equation (25). In the CT model (left turn), the transmitter’s turn rate is set as w=−5 degrees per second in Equation (25). In addition, the *model transition probability* is selected as

(28) $$\begin{array}{c} p_{ij}=\left(\begin{array}{ccc} 0.9 & 0.05 & 0.05\\ 0.1 & 0.8 & 0.1\\ 0.05 & 0.15 & 0.8 \end{array}\right). \end{array}$$

The transmitter’s movement model is selected as

(29) $$\begin{array}{ccc} E_{k}\left[1\right] & = & E_{k-1}\left[1\right]+V_{k-1}^{tgt}\times \cos\left(\theta_{k-1}\right)\times T,\\ E_{k}\left[1\right] & = & E_{k-1}\left[2\right]+V_{k-1}^{tgt}\times \sin\left(\theta_{k-1}\right)\times T,\\ \theta_{k} & = & \theta_{k-1}+rate_{k-1}^{a}\times T,\\ V_{k}^{tgt} & = & V_{k-1}^{tgt}+rate_{k-1}^{s}\times T. \end{array}$$

Here, $E_{k}=\left(E_{k}\left[1\right],E_{k}\left[2\right]\right)$ is the transmitter location at sample-stamp *k*. $\theta_{k}$ indicates the orientation angle of the transmitter at sample-stamp *k*. $V_{k}^{tgt}$ is the speed of the transmitter at sample-stamp *k*. $rate_{k}^{a}$ is the orientation change rate at sample-stamp *k*. $rate_{k}^{s}$ is the speed change rate at sample-stamp *k*.

### 5.1. Monte Carlo (MC) Simulations

We ran $M_{c}=100$ MC simulations to rigorously demonstrate the performance of our tracking approach. Let ${\hat{E}}_{k}^{t}$, where $t\in \{1,2,\ldots ,M_{c}\}$ define the transmitter estimate at sample-stamp *k* utilizing the *t*-th MC simulation. This article applies ${\hat{E}}_{k}^{t}=\left[{\hat{X}}_{k|k}\left[1\right],{\hat{X}}_{k|k}\left[2\right]\right]$.

Under each MC simulation, M=8 receivers are randomly deployed in the obstacle-free space inside the 2D workspace. While the transmitter moves in the workspace, an LOS receiver may become an NLOS receiver, and vice versa.

The following RMSE (in meters) is applied:

(30) $$\begin{array}{c} RMSE_{k}=\sqrt{\frac{\sum_{t=1}^{M_{c}}{∥{\hat{E}}_{k}^{t}-E_{k}∥}^{2}}{M_{c}}}. \end{array}$$

Here, $E_{k}$ is the true transmitter location at sample-stamp *k*.

We show the outperformance of the proposed tracking filter by comparing it with filters in [42,43,44]. Since [42,43,44] did not consider the tracking of a moving transmitter, the transmitter estimate of [42,43,44] was derived at each sample-step. IMM KF was not applied for a transmitter estimate of [42,43,44].

In the following figures related to $RMSE_{k}$, [Pro] indicates $RMSE_{k}$, as we apply the proposed estimate $\hat{E}$ in Equation (23). IMM[Pro] indicates $RMSE_{k}$ for the proposed IMM KF, whose measurements are given by $\hat{E}$ in Equation (23). [Su] indicates $RMSE_{k}$ for [42]. [Yang] indicates $RMSE_{k}$ for [43]. [Apo] indicates $RMSE_{k}$ for [44].

### 5.2. Scenario 1

Figure 1 plots the 2D obstacle environment considered in the simulations. The transmitter location at each sample-stamp is plotted with a red cross. The start point of the transmitter is marked with a black circle, and the end point of the transmitter is marked with a black diamond. Reflected signals can be generated due to obstacles, which are plotted with rectangles in the workspace. As the transmitter moves, an LOS receiver may become an NLOS receiver, and vice versa. At the moment when the simulation ends, the LOS receivers are plotted with green asterisks, and NLOS receivers are plotted with black asterisks.

In Figure 1, the LOS between the transmitter and an LOS receiver is not blocked by an obstacle. However, LOS between the transmitter and an NLOS receiver is blocked by an obstacle. Since the true transmitter location is not accessible, we do not know which receivers are LOS receivers.

In Figure 1, the transmitter maneuvers as follows. Initially, the transmitter’s speed $V_{0}^{tgt}$ is 8 m/s. At sample-stamp 0, the transmitter’s location $E_{0}$ is (500, 3200). From 50 to 100 s, the transmitter varies its speed with a change rate of $rate_{k}^{s}=-0.1$ m/s^2^. From 150 to 180 s, the transmitter varies its orientation with a change rate of $rate_{k}^{a}=-3$ degrees per second. The simulation is finished after 300 s have elapsed.

**Figure 2:**
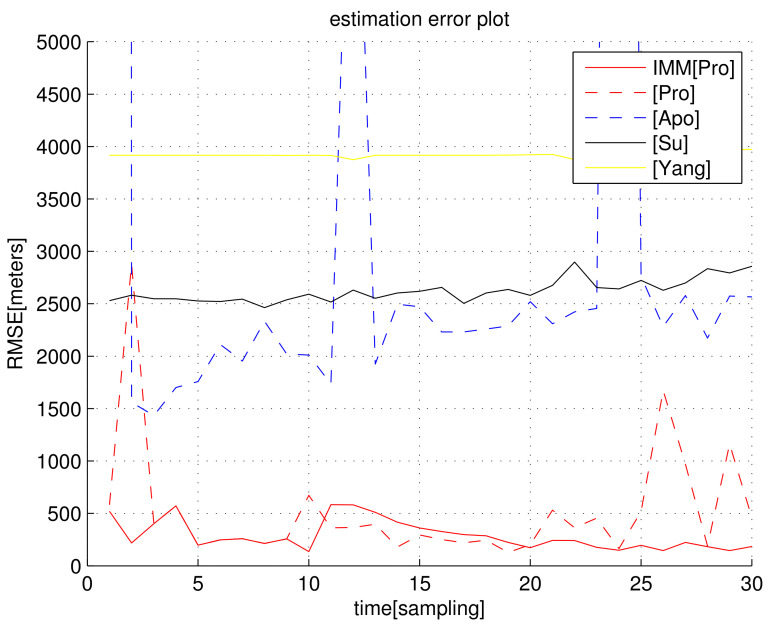
$RMSE_{k}$ with respect to sample-stamp *k* (scenario 1). We set σ=5/C s, which implies that the distance noise in LOS measurements is 5 m. The proposed filters ([Pro] and IMM[Pro]) outperform all other location methods.

For the scenario in Figure 1, Figure 2 plots $RMSE_{k}$ as *k* varies. We set σ=5/C s, which implies that the distance noise in LOS measurements is 5 m. In general, $RMSE_{k}$ under the proposed filters IMM[Pro] and [Pro] decreases as *k* increases. Figure 2 shows that the proposed filters ([Pro] and IMM[Pro]) outperform all other location methods, considering the estimation accuracy.

Figure 2 shows that, as we apply the IMM KF, the RMSE decreases compared to the case where the IMM KF is not applied. In the case where we use [Pro] (IMM KF is not used), Assumption (A1) may not hold depending on the deployed receiver positions. This leads to an overshoot in the RMSE for [Pro], as plotted in Figure 2. In IMM[Pro], this overshoot is mitigated since the IMM KF can discard false TDOA measurements using (27).

Considering the simulation in Figure 2, the computational load (running time of one MC simulation) of each algorithm is analyzed in Table 1. An SDP approach in [42,43] utilized the optimization tool; hence, its computational load is much higher than in non-optimization methods. Considering both computational load and localization accuracy, IMM[Pro] and [Pro] outperform all other location methods.

The sampling interval is T=10 s, and the entire scenario runs for 30 sampling-steps. Thus, the entire scenario runs for 300 s. Under the proposed methods, the simulation running time of one scenario is only 2 s. Thus, we argue that the proposed methods are suitable for real-time target tracking.

### 5.3. Scenario 2

**Figure 3:**
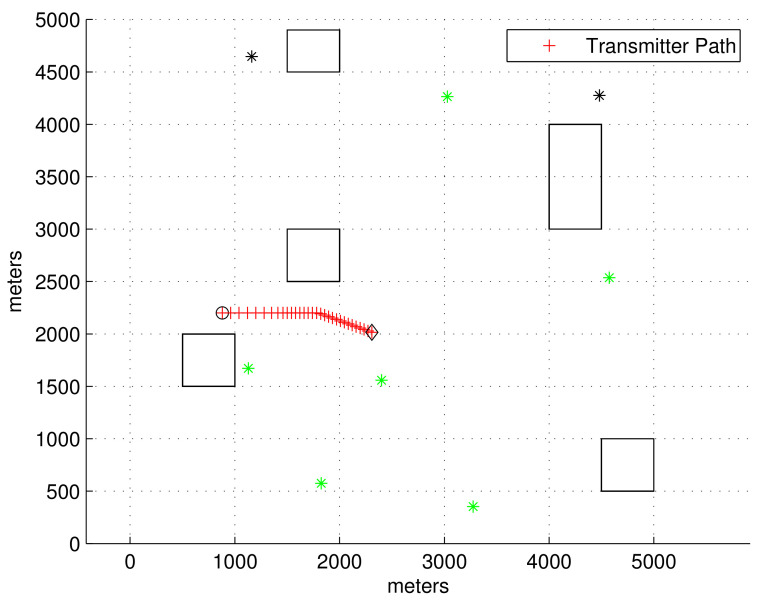
Scenario 2. The transmitter’s position at every sample-stamp is plotted with a red cross. The start point of the transmitter is marked with a black circle, and the end point of the transmitter is marked with a black diamond. Reflected signals can be generated due to obstacles, which are plotted with rectangles in the workspace. As the transmitter moves, an LOS receiver may become an NLOS receiver, and vice versa. At the moment when the simulation ends, LOS receivers are plotted with green asterisks, and NLOS receivers are plotted with black asterisks.

Figure 3 plots the obstacle environment considered in the second scenario. In the figure, the transmitter’s position at every sample-stamp is plotted with a red cross. The start point of the transmitter is marked with a black circle, and the end point of the transmitter is marked with a black diamond. Reflected signals can be generated due to obstacles, which are plotted with rectangles in the workspace. As the transmitter moves, an LOS receiver may become an NLOS receiver, and vice versa. At the moment when the simulation ends, the LOS receivers are plotted with green asterisks, and NLOS receivers are plotted with black asterisks.

In Scenario 2, the transmitter maneuvers as follows. Initially, the transmitter’s speed $V_{0}^{tgt}$ is 8 m/s. At sample-stamp 0, the transmitter’s location $E_{0}$ is (1000, 2200). From 50 to 100 s, the transmitter varies its speed with a change rate of $rate_{k}^{s}=-0.1$ m/s^2^. From 150 to 180 s, the transmitter varies its orientation with a change rate of $rate_{k}^{a}=-1$ degree per second. The simulation is finished after 300 s have elapsed.

**Figure 4:**
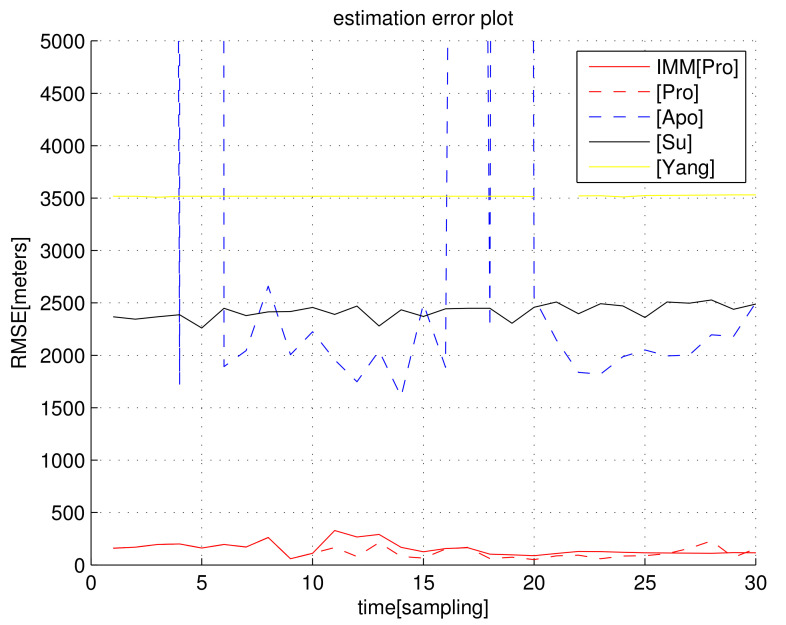
$RMSE_{k}$ with respect to sample-stamp *k* (scenario 2). We set σ=5/C s, which implies that the distance noise in LOS measurements is 5 m. The proposed filters ([Pro] and IMM[Pro]) outperform all other location methods, considering the localization accuracy.

In the scenario of Figure 3, Figure 4 shows $RMSE_{k}$ as *k* varies. We set σ=5/C s, which implies that the distance noise in LOS measurements is 5 m. The proposed filters ([Pro] and IMM[Pro]) outperform all other location methods.

**Figure 5:**
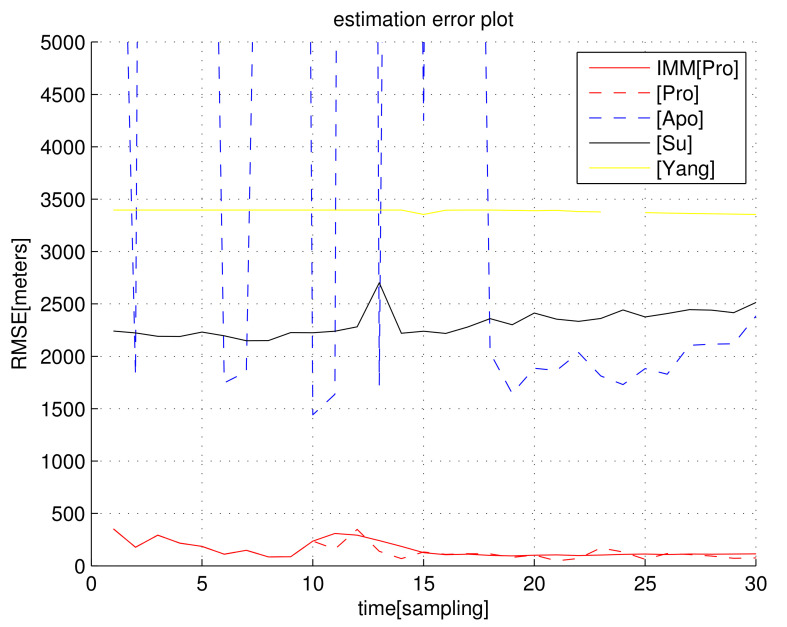
$RMSE_{k}$ with respect to sample-stamp *k* (scenario 2). We set σ=10/C s. The proposed filters ([Pro] and IMM[Pro]) outperform all other location methods.

In the scenario of Figure 3, Figure 5 shows $RMSE_{k}$ as *k* varies. We set σ=10/C s, which implies that the distance noise in LOS measurements is 10 m. The proposed filters ([Pro] and IMM[Pro]) outperform all other location methods, considering localization accuracy. In general, as one utilizes the IMM KF, the RMSE decreases compared to cases where the IMM KF is not applied.

Considering the simulation in Figure 5, the computational load (running time of one MC simulation) of each algorithm is analyzed in Table 2. An SDP approach in [42,43] utilized the optimization tool; hence, its computational load is much higher than in non-optimization methods. Considering both the computational load and localization accuracy, IMM[Pro] and [Pro] outperform all other methods.

## 6. Conclusions

Considering cluttered, unknown mixed LOS/NLOS environments, this article is unique in tracking a moving transmitter while decreasing NLOS error in TDOA localization. This paper proposes an algorithm to locate a transmitter while decreasing NLOS error in TDOA-only measurements. For tracking a moving transmitter in real time, this article integrated the proposed localization algorithm with the IMM KF.

Since the proposed location filter runs fast, it can be applied to track a moving transmitter in real time. The superiority of our transmitter estimate approach was demonstrated by comparing it with other state-of-the-art TDOA methods [42,43,44] utilizing MATLAB simulations.

We further integrated the proposed localization algorithm and the IMM KF to track a moving transmitter in real time. MATLAB simulations showed that, as we applied the IMM KF, the RMSE decreased compared to the case where the IMM KF was not applied. In the future, we will conduct experiments utilizing real receivers to demonstrate the performance of the proposed approach in practice.

Note that our tracking filter works regardless of the movement of receivers. We only required that the receivers be localized in global coordinate systems and that communication links among receivers be established. As long as the receivers were localized, we could locate the transmitter by applying the proposed filter to the TDOA measurements of all receivers.

## Figures and Tables

**Table 1:** Computational load analysis (simulation of Figure 2).

Alg.	OneMCTime
IMM[Pro]	2 s
[Pro]	2 s
[Apo]	4 s
[Su]	78 s
[Yang]	35 s

**Table 2:** Computational load analysis (simulation of Figure 5).

Alg.	OneMCTime
IMM[Pro]	3 s.
[Pro]	3 s.
[Apo]	6 s.
[Su]	74 s.
[Yang]	48 s.

## Data Availability

Not applicable.
